# Lifestyle factors and BMI attenuate relationships between biomarkers of inflammation and depressive symptoms and well-being: A cross-sectional study

**DOI:** 10.1016/j.bbih.2024.100759

**Published:** 2024-03-18

**Authors:** Seán R. Millar, Janas M. Harrington, Ivan J. Perry, Catherine M. Phillips

**Affiliations:** a School of Public Health, University College Cork, Cork, Ireland; b School of Public Health, Physiotherapy and Sports Science, University College Dublin, Dublin 4, Ireland

**Keywords:** Depression, Well-being, Chronic inflammation, Lifestyle factors

## Abstract

**Background:**

Mental disorders are a growing public health concern and evidence has linked chronic low-grade inflammation with depression and well-being. Research also suggests that certain modifiable lifestyle factors such as smoking, alcohol use, physical activity, diet quality and BMI are related to psychological health. These may modulate the relationship between low-grade inflammation and mental health conditions. In this study we examined inflammatory biomarker associations with two psychological health scores and investigated whether relationships are influenced by lifestyle factors and BMI.

**Methods:**

This was a cross-sectional study of 1824 middle-to older-aged men and women randomly selected from a large primary care centre. Depressive symptoms and well-being were assessed using the 20-item Centre for Epidemiologic Studies Depression (CES-D) Scale and the World Health Organization-Five (WHO-5) Well-Being Index. Linear regression analyses were performed to examine depression and well-being score relationships with six inflammatory biomarkers, and a composite inflammatory biomarker score, adjusting for demographic characteristics, health conditions, lifestyle factors and BMI.

**Results:**

Depression and well-being score associations with complement component 3 (CES-D only) c-reactive protein, interleukin 6, leptin, white blood cell counts, neutrophils and the inflammatory biomarker score were observed. These relationships survived adjustment for demographic variables and health conditions but were attenuated in models which included lifestyle factors and BMI. In final models, only leptin (β = 0.566, *p* = 0.018) and inflammatory score (β = 0.137, *p* = 0.004) associations with the CES-D score remained.

**Conclusions:**

These findings suggest that the relationship between systemic low-grade inflammation and depressive symptoms and well-being may be largely explained by lifestyle factors and adiposity, highlighting the potential importance of promoting a healthy lifestyle in the treatment of depressive disorders.

## Highlights

• Mental health score associations with pro-inflammatory biomarkers were observed.
• These relationships survived adjustment for demographic variables and health conditions.
• Associations were attenuated in models which adjusted for lifestyle factors and BMI.
• The relationship between systemic low-grade inflammation and mental health may be largely explained by modifiable lifestyle factors.

## 1 Introduction

Depressive disorders are a growing public health concern with an estimated 300 million people around the world afflicted (Herrman et al., 2019). Data from the Global Burden of Disease Study demonstrate the notable contribution of mental health and depressive disorders to the global burden of non-communicable diseases through years lived with disability (Collaborators, 2022), which in terms of disease burden, is currently the leading cause of disability worldwide (Donoso et al., 2023).

Increasing evidence has linked chronic low-grade inflammation and raised immune activation with depression and well-being (Berk et al., 2013; Osimo et al., 2019). Although some studies have reported inconsistent results (Gialluisi et al., 2020; Miller, 2020), a meta-analysis of 37 studies which examined the prevalence of low-grade inflammation in depression, using different c-reactive protein (CRP) levels, found that approximately one-in-four patients with depression showed CRP levels >3 mg/l, while three patients out of five had mildly elevated CRP (>1 mg/l) levels (Osimo et al., 2019). Research has also associated other pro-inflammatory biomarkers with mental health disorders and complement component 3 (C3), interleukin 6 (IL-6), leptin and white blood cell count (WBC) concentrations are reported to be higher in subjects with depression (Cernea et al., 2019; Luo et al., 2022; Shafiee et al., 2017; Ting et al., 2020).

Inflammatory biomarkers and their downstream signalling pathways are though to decrease the synthesis and release, and increase the reuptake, of serotonin, norepinephrine and dopamine, with the effects of inflammation on neurotransmitter systems ultimately affecting neurocircuitry (Miller, 2020). Studies over the last decade have additionally linked low-grade systemic inflammation with chronic conditions, including type 2 diabetes, cardiovascular disease and many cancers (Donath and Shoelson, 2011; Hansson and Hermansson, 2011; Howcroft et al., 2013; Millar et al., 2015; Phillips and Perry, 2013), which in turn can lead to an increase in depressive symptoms and poorer well-being (Maher et al., 2016).

It is well established that certain modifiable lifestyle factors are related to mental health. Using data from the third national Survey of Lifestyle, Attitudes and Nutrition (SLÁN) in Ireland (n = 10,364) Harrington et al. showed moderate alcohol intake, being physically active and higher dietary quality to be associated with better mental health among free-living individuals aged 18 years and over (Harrington et al., 2009). Another study demonstrated that a combination of healthy lifestyle behaviours (never smoker, moderate alcohol consumption, moderate or high levels of physical activity and healthy diet) was associated with psychological health in middle-to older-aged adults; subjects with the fewest healthy behaviours (zero or one) were twice as likely to have depression compared to those with four (Maher et al., 2016).

Relationships between lifestyle factors and circulating inflammatory biomarkers have also been observed (Millar et al., 2021, 2022a, 2022c), with research showing tobacco use to be related to higher levels of IL-6, WBCs, and white blood cell constituents, and alcohol use to be inversely related to IL-6 concentrations. Low-level physical activity has been found to be positively associated with CRP, IL-6 and immune cell levels, while higher diet quality has been observed to be associated with reduced IL-6 and WBC concentrations. In addition, research has demonstrated adiposity defined by body mass index (BMI) to show the greatest number of significant relationships with inflammatory biomarkers after adjustment for other modifiable factors (Millar et al., 2022a).

Anti-depressant medications currently used in clinical practice exclusively target serotonin and/or noradrenaline pathways (Hillhouse and Porter, 2015). However, in addition to anti-depressant medications, there is evidence to suggest that a number of adjunctive therapies, including exercise and dietary intervention, are beneficial because of their anti-inflammatory actions (Donoso et al., 2023). Modifiable risk factors may modulate the relationship between low-grade inflammation and mental health and lifestyle therapies could help reduce metabolic dysfunction and chronic disease risk (Millar et al., 2022b). Therefore, using a random sample of 1824 middle-to older-aged men and women, the aim of this study is twofold: (1) to examine relationships between inflammatory biomarkers, an inflammatory biomarker score, and two psychological health scores and (2) to investigate whether these relationships are influenced by lifestyle factors and BMI.

## 2 Materials and methods

### 2.1 Study population and setting

This study is a secondary analysis of data collected from the Cork and Kerry Diabetes and Heart Disease Study (Phase II – Mitchelstown Cohort). Full details of the study, which aimed to examine major cardiovascular disease risk factors in a middle-to older-aged population, have been described previously (Kearney et al., 2013). In brief, the Mitchelstown Cohort was a single-centre study conducted between 2010 and 2011. A random sample was recruited from a large primary care centre in Mitchelstown, County Cork, Ireland. The Living Health Clinic serves a population of approximately 20,000 predominantly White European subjects, with a mix of urban and rural residents. Stratified sampling was employed to recruit equal numbers of men and women from all registered attending patients in the 45–70-year age group. In total, 3807 potential participants were selected from the practice list. Following the exclusion of duplicates, deaths and subjects incapable of consenting or attending appointment, 3051 were invited to participate in the study and of these, about two-thirds (2,047, 49% male) completed the questionnaire and physical examination components of the baseline assessment. Ethics committee approval conforming to the Declaration of Helsinki was obtained from the Clinical Research Ethics Committee of University College Cork. A letter signed by the contact GP in the clinic was sent out to all selected participants with a reply slip indicating acceptance or refusal. All participants gave signed informed consent, including permission to use their data for research purposes.

### 2.2 Clinical procedures

Study participants attended the clinic in the morning after an overnight fast and blood samples were taken on arrival. Fasting glucose and glycated haemoglobin A_1c_ (HbA_1c_) concentrations were measured in fresh samples by Cork University Hospital Biochemistry Laboratory using standardised procedures. Glucose concentrations were determined using a glucose hexokinase assay (Olympus Life and Material Science Europa Ltd., Lismeehan, Co. Clare, Ireland) and HbA_1c_ levels were measured in the haematology laboratory on an automated high-pressure liquid chromatography instrument Tosoh G7 [Tosoh HLC-723 (G7), Tosoh Europe N.V, Tessenderlo, Belgium]. CRP, IL-6 and leptin were assessed using a biochip array system (Evidence Investigator; Randox Laboratories, UK). C3 was measured by immunoturbidimetric assay (RX Daytona; Randox Laboratories). WBC and neutrophil concentrations were determined by flow cytometry technology as part of a full blood count.

Anthropometric measurements were performed by trained researchers with reference to a standard operating procedures manual. Height was measured with a portable Seca Leicester height/length stadiometer (Seca, Birmingham, UK) and weight was measured using a portable electronic Tanita WB-100MA weighing scale (Tanita Corp, IL, USA). The weighing scale was placed on a firm flat surface and was calibrated weekly. BMI was calculated as weight in kilograms divided by the square of height in meters.

### 2.3 Data collection

A general health and lifestyle questionnaire assessed demographic variables, lifestyle behaviours and morbidity. Information on age, sex, education, prescription anti-inflammatory medication use, smoking status, alcohol use, diagnosis of type 2 diabetes and cancer was provided by participants. The presence of cardiovascular disease was obtained by asking study participants if they had been diagnosed with any one of the following seven conditions: Heart Attack (including coronary thrombosis or myocardial infarction), Heart Failure, Angina, Aortic Aneurysm, Hardening of the Arteries, Stroke or any other Heart Trouble. Subjects who indicated a diagnosis of any one of these conditions were classified as having cardiovascular disease. Physical activity levels were measured using the validated International Physical Activity Questionnaire (IPAQ) (Craig et al., 2003). Depressive symptoms and well-being were assessed from participants’ self-completed questionnaires using the 20-item Centre for Epidemiologic Studies Depression (CES-D) Scale (Radloff, 1977), designed to evaluate the frequency and severity of depressive symptoms, and the World Health Organization-Five (WHO-5) Well-Being Index (Organization, 1998). Following exclusion of individuals without mental health data (n = 223), the remaining 1824 participants were used in the analyses.

### 2.4 Dietary assessment

A Food Frequency Questionnaire (FFQ) was used for dietary assessment. Diet was evaluated using a modified version of the self-completed European Prospective Investigation into Cancer and Nutrition (EPIC) FFQ (Riboli et al., 1997), which has been validated extensively in several populations (Bingham et al., 1997). Adapted to reflect the Irish diet, the 150-item semi-quantitative FFQ used in the current study was originally validated for use in the Irish population using food diaries and a protein biomarker in a volunteer sample (Harrington, 1997) and incorporated into the SLÁN Irish National Surveys of Lifestyle Attitudes and Nutrition (1998), 2002, 2007; Morgan et al. (2008). The average medium serving of each food item consumed by participants over the last 12 months was converted into quantities using standard portion sizes. Food item quantity was expressed as (g/d) and beverages as (ml/d).

Based on the FFQ, the DASH score was constructed. DASH is a dietary pattern rich in fruits, vegetables, whole grains and low-fat dairy foods and is limited in sugar-sweetened foods and beverages, red meat and added fats. This diet has been promoted by the National Heart, Lung and Blood Institute (part of the National Institutes of Health, a United States government organisation) to prevent and control hypertension. For each food group, consumption was divided into quintiles, and participants were classified according to their intake ranking. Component scores were summed and an overall DASH score was calculated for each person. DASH diet scores ranged from 11 to 41. Lower scores represent poorer and higher scores represent better quality diet (Harrington et al., 2013).

### 2.5 Classification and scoring of variables

Categories of education included ‘some primary (not complete)’, ‘primary or equivalent’, ‘intermediate/group certiﬁcate or equivalent’, ‘leaving certiﬁcate or equivalent’, ‘diploma/certiﬁcate’, ‘primary university degree’ and ‘postgraduate/higher degree’. These were collapsed and recoded into a dichotomous variable: ‘primary education only’ (finished full-time education at 13 years or younger) and ‘intermediate or higher’.

Smoking status was deﬁned as follows: (i) never smoked, i.e. having never smoked at least 100 cigarettes (5 packs) in their entire life; (ii) former smoker, i.e. having smoked 100 cigarettes in their entire life and do not smoke at present; and (iii) current smoker, i.e. smoking at present. These deﬁnitions were the same as those used in the SLÁN National Health and Lifestyle Survey (Harrington et al., 2008). A binary variable was then created: ‘never/former smoker’ or ‘current smoker’. Alcohol consumption was measured in units of alcohol consumed on a weekly basis and was categorised into the following levels: (i) non-drinker, i.e. <1 drink per week; (ii) moderate drinker, i.e. between 1 and 14 drinks per week; and (iii) heavy drinker, i.e. >14 drinks per week. Moderate drinker was deﬁned on the basis of previous work from the EPIC in the United Kingdom by Khaw et al. (2008). For the current analysis, these were then re-categorised as ‘moderate/non-drinker’ or ‘heavy drinker’. Physical activity was categorised as low, moderate and high levels of activity using the IPAQ. This was then recoded as a dichotomous variable: ‘moderate/high’ or ‘low’ physical activity. We classified poor diet quality as a DASH diet score in the bottom 60% for the study sample (Millar et al., 2022a). High BMI was defined as a BMI ≥25 kg/m^2^.

Type 2 diabetes was determined as a fasting glucose level ≥7.0 mmol/l or HbA_1c_ level ≥6.5% (≥48 mmol/mol) (Association, 2019) or by self-reported diagnosis. A composite inflammatory score was calculated based on quartiles of C3, CRP, IL-6, leptin, WBCs and neutrophils. Quartiles 1–4 for each biomarker (where 1 indicates the least pro-inflammatory level and 4 the most pro-inflammatory level) were summed. Scores ranged from 1 to 24.

### 2.6 Statistical analysis

Descriptive characteristics were examined according to depression and well-being score quartiles. Categorical features are presented as numbers (n) and percentages (%) and continuous variables are shown as a mean, plus or minus one standard deviation (±SD) or a median and interquartile range (IQR). Differences across CES-D and WHO-5 score quartiles were analysed using a Pearson's chi-square test for categorical variables, an ANOVA for continuous data that followed a normal distribution or a Kruskal-Wallis test for skewed variables. In order to improve model fit and reduce outlier influence, skewed biomarker data (CRP, IL-6, leptin, WBC and neutrophils) were log-transformed and linear regression analyses were performed to determine inflammatory biomarker associations with depression and well-being scores. Three models were run: a crude model; a second model which adjusted for demographic characteristics (age, sex and education) and a third model which additionally adjusted for anti-inflammatory medication use, type 2 diabetes, cardiovascular disease and cancer. Further analyses were conducted which adjusted for lifestyle factors and BMI, both individually and all together.

To quantify the influence of demographic characteristics, health conditions, lifestyle factors and BMI, in each adjusted model we calculated R^2^ values from dominance analyses. Dominance analysis determines the importance of independent variables in multiple regression models by comparing their additional R^2^ contributions across all subset models (Azen and Budescu, 2003). Data analysis was conducted using Stata SE Version 13 (Stata Corporation, College Station, TX, USA) for Windows. Dominance analyses were performed using the **domir** package in R (Luchman, 2022). For all analyses, a *p* value (two-tailed) of less than 0.05 was considered to indicate statistical significance.

## 3 Results

### 3.1 Descriptive characteristics

Table 1 shows the characteristics of study participants according to CES-D and WHO-5 score quartiles. Higher CES-D scores indicate more severe depressive symptoms whereas higher WHO-5 scores indicate greater well-being. Participants with more severe depressive symptoms (quartile 4 compared to quartile 1 for the CES-D) and poorer well-being (quartile 1 compared to quartile 4 for the WHO-5) were less likely to be male and were more likely to have type 2 diabetes and to have low levels of physical activity. Significant differences in inflammatory biomarker concentrations were observed across both CES-D and WHO-5 score quartiles.

**Table 1:** Descriptive characteristics and inflammatory profiles of study participants according to depression and well-being score quartiles (n = 1824).

Variable	**CES-D score**				
	**Q1** (n = 551)	**Q2** (n = 430)	**Q3** (n = 441)	**Q4** (n = 402)	*p*
Age in years (median, IQR)	59.0 (55.0, 64.0)	58.0 (54.0, 63.0)	59 (54.0, 64.0)	58.0 (54.0, 62.0)	0.038^c^
Male (n, %)	297 (53.9)	202 (47.0)	226 (51.2)	177 (44.0)	0.013^a^
Primary education only (n, %)	134 (25.6)	94 (23.3)	117 (27.9)	108 (28.4)	0.335^a^
On anti-inflammatory medications (n, %)	86 (15.8)	57 (13.4)	82 (18.8)	75 (18.9)	0.097^a^
Type 2 diabetes (n, %)	37 (6.7)	30 (7.0)	32 (7.3)	59 (14.7)	<0.001^a^
Cardiovascular disease (n, %)	59 (10.7)	35 (8.1)	52 (11.8)	42 (10.4)	0.342^a^
Cancer (n, %)	21 (3.8)	13 (3.0)	24 (5.4)	13 (3.2)	0.243^a^
Current smoker (n, %)	69 (12.6)	55 (12.9)	73 (16.6)	66 (16.5)	0.147^a^
Heavy drinker (n, %)	294 (53.4)	240 (55.8)	224 (50.8)	213 (53.0)	0.528^a^
Low-level physical activity (n, %)	222 (42.3)	170 (40.6)	199 (47.5)	226 (58.1)	<0.001^a^
Poor diet quality (n, %)	320 (60.0)	272 (64.9)	249 (61.6)	240 (63.7)	0.431^a^
High BMI (n, %)	430 (78.0)	325 (75.6)	359 (81.4)	322 (80.7)	0.137^a^
C3, mg/dl (mean ± SD)	133.75 ± 22.8	132.57 ± 25.7	137.67 ± 24.4	138.56 ± 24.8	<0.001^b^
CRP, ng/ml (median, IQR)	1.28 (0.94, 2.03)	1.29 (0.93, 1.97)	1.34 (0.99, 2.52)	1.42 (0.99, 2.37)	0.012^c^
IL-6, pg/ml (median, IQR)	1.74 (1.18, 2.77)	1.61 (1.08, 2.72)	1.75 (1.16, 2.89)	1.90 (1.28, 3.07)	0.017^c^
Leptin, ng/ml (median, IQR)	1.75 (0.99, 2.85)	1.62 (1.00, 2.83)	2.00 (1.24, 3.33)	2.07 (1.28, 3.32)	0.001^c^
WBC, 10^9^/l (median, IQR)	5.70 (4.80, 6.70)	5.50 (4.60, 6.60)	5.80 (5.00, 7.00)	5.80 (4.70, 7.00)	0.013^c^
Neutrophils, 10⁹/l (median, IQR)	3.11 (2.52, 3.86)	3.03 (2.40, 3.84)	3.18 (2.59, 4.04)	3.19 (2.46, 4.12)	0.032^c^
Inflammatory score (mean ± SD)	14.41 ± 4.5	14.00 ± 4.8	15.09 ± 4.4	15.42 ± 4.7	<0.001^b^
	**WHO-5 score**				
	**Q1** (n = 510)	**Q2** (n = 450)	**Q3** (n = 484)	**Q4** (n = 380)	*p*
Age in years (median, IQR)	57.0 (53.0, 62.0)	58.0 (54.0, 62.0)	58.0 (54.0, 63.0)	62.0 (57.0, 66.0)	<0.001^c^
Male (n, %)	230 (45.1)	228 (50.7)	236 (48.8)	208 (54.7)	0.037^a^
Primary education only (n, %)	129 (26.7)	93 (21.6)	116 (25.4)	115 (32.2)	0.009^a^
On anti-inflammatory medications (n, %)	79 (15.7)	71 (16.0)	85 (17.7)	65 (17.4)	0.799^a^
Type 2 diabetes (n, %)	59 (11.6)	33 (7.3)	36 (7.4)	30 (7.9)	0.054^a^
Cardiovascular disease (n, %)	50 (9.8)	51 (11.3)	46 (9.5)	41 (10.8)	0.781^a^
Cancer (n, %)	21 (4.1)	15 (3.3)	20 (4.1)	15 (3.9)	0.914^a^
Current smoker (n, %)	78 (15.3)	74 (16.5)	65 (13.5)	46 (12.2)	0.3^a^
Heavy drinker (n, %)	284 (55.7)	260 (58.8)	257 (53.1)	170 (44.7)	0.001^a^
Low-level physical activity (n, %)	271 (55.4)	205 (46.9)	213 (45.4)	128 (35.9)	<0.001^a^
Poor diet quality (n, %)	309 (64.8)	273 (62.9)	281 (61.4)	218 (59.9)	0.496^a^
High BMI (n, %)	410 (80.9)	337 (74.9)	379 (78.3)	310 (81.6)	0.064^a^
C3, mg/dl (mean ± SD)	138.15 ± 25.2	133.84 ± 25.6	134.91 ± 22.6	134.60 ± 24.0	0.033^b^
CRP, ng/ml (median, IQR)	1.43 (0.99, 2.50)	1.24 (0.92, 1.93)	1.27 (0.95, 2.17)	1.37 (0.98, 2.22)	0.004^c^
IL-6, pg/ml (median, IQR)	1.87 (1.20, 3.14)	1.64 (1.13, 2.66)	1.69 (1.14, 2.79)	1.80 (1.24, 2.83)	0.033^c^
Leptin, ng/ml (median, IQR)	2.05 (1.22, 3.37)	1.91 (1.08, 3.00)	2.00 (1.08, 2.98)	1.59 (0.99, 2.64)	0.001^c^
WBC, 10^9^/l (median, IQR)	5.90 (4.80, 7.10)	5.70 (4.80, 7.00)	5.60 (4.80, 6.50)	5.60 (4.70, 6.70)	0.044^c^
Neutrophils, 10⁹/l (median, IQR)	3.20 (2.49, 4.15)	3.11 (2.51, 3.90)	3.09 (2.54, 3.81)	3.10 (2.50, 3.91)	0.247^c^
Inflammatory score (mean ± SD)	15.41 ± 4.9	14.45 ± 4.5	14.33 ± 4.6	14.51 ± 4.3	0.001^b^

Abbreviations: C3: complement component 3; CES-D: Centre for Epidemiologic Studies Depression; CRP: c-reactive protein; IL-6: interleukin 6; WBC: white blood cell count.

*p* determined from:

a Pearson's chi-square test.

b ANOVA.

c Kruskal-Wallis test.

### 3.2 Linear regression

Linear regression analyses describing associations between CES-D and WHO-5 scores and inflammatory biomarkers are shown in Table 2. Depression and well-being score associations with C3 (CES-D only) CRP, IL-6, leptin, WBCs, neutrophils and the inflammatory score were observed, with the CES-D score being positively associated, and the WHO-5 score being inversely associated, with biomarker concentrations and the inflammatory biomarker score. These relationships survived adjustment for demographic characteristics and health conditions, with health conditions explaining 1.3%–1.6% and 0.6% of the variance of the CES-D score and WHO-5 score, respectively.

**Table 2:** Linear regression analysis of the associations between inflammatory biomarkers and depression and well-being scores.

Depression/well-being scores	C3		CRP		IL-6		Leptin		WBC		Neutrophils		Inflammatory score	
	β (R^2^)	*P*	β (R^2^)	*P*	β (R^2^)	*P*	β (R^2^)	*P*	β (R^2^)	*P*	β (R^2^)	*P*	β (R^2^)	*P*
**CES-D score**														
Model 1	0.027 (0.007)	**<0.001**	0.880 (0.006)	**0.001**	0.675 (0.004)	**0.007**	0.841 (0.009)	**<0.001**	1.856 (0.004)	**0.006**	1.582 (0.005)	**0.004**	0.203 (0.014)	**<0.001**
Model 2	0.023 (0.013)	**0.003**	0.814 (0.012)	**0.003**	0.920 (0.015)	**<0.001**	0.726 (0.012)	**0.001**	2.142 (0.014)	**0.002**	1.869 (0.014)	**0.001**	0.208 (0.014)	**<0.001**
Model 3	0.018 (0.013)	**0.018**	0.797 (0.015)	**0.004**	0.805 (0.016)	**0.002**	0.682 (0.015)	**0.002**	1.464 (0.015)	**0.037**	1.400 (0.015)	**0.014**	0.190 (0.016)	**<0.001**

**WHO-5 score**														
Model 1	−0.009 (0.002)	0.075	−0.449 (0.003)	**0.012**	−0.207 (0.001)	0.22	−0.459 (0.006)	**0.001**	−1.191 (0.003)	**0.009**	−0.895 (0.003)	**0.015**	−0.097 (0.007)	**<0.001**
Model 2	−0.008 (0.045)	0.117	−0.488 (0.045)	**0.008**	−0.552 (0.047)	**0.002**	−0.342 (0.044)	**0.017**	−1.428 (0.046)	**0.002**	−1.148 (0.045)	**0.002**	−0.110 (0.045)	**<0.001**
Model 3	−0.006 (0.006)	0.251	−0.473 (0.006)	**0.011**	−0.509 (0.006)	**0.004**	−0.325 (0.006)	**0.025**	−1.080 (0.006)	**0.022**	−0.904 (0.006)	**0.018**	−0.104 (0.006)	**<0.001**

Abbreviations: C3: complement component 3; CES-D: Centre for Epidemiologic Studies Depression; CRP: c-reactive protein; IL-6: interleukin 6; WBC: white blood cell count.

Model 1: univariate.

Model 2: adjusted for demographic characteristics (age, sex and education).

Model 3: additionally adjusted for health conditions (anti-inflammatory medication use, type 2 diabetes, cardiovascular disease and cancer).

Unstandardised β coefficients are shown. CRP, IL-6, leptin, WBC and neutrophils are log-transformed. Significant *p* shown in **bold**. R^2^ in adjusted models determined from dominance analyses. R^2^ in model 2 represents the variance explained by demographic characteristics; R^2^ in model 3 represents the variance explained by health conditions.

Linear regression models demonstrating associations between inflammatory biomarkers and depression/well-being scores, which additionally adjusted for lifestyle factors and BMI, are shown in Table 3. Among individual factors, low-level physical activity was found to explain a greater variance of both CES-D and WHO-5 scores, with models adjusting for physical activity demonstrating greater attenuation than models which adjusted for other variables. However, models which adjusted for all lifestyle factors and BMI in general showed the greatest attenuation. In final models, only leptin (β = 0.566, *p* = 0.018) and inflammatory score (β = 0.137, *p* = 0.004) associations with the CES-D score remained.

**Table 3:** Linear regression analysis of the associations between inflammatory biomarkers and depression and well-being scores, adjusted for lifestyle factors and BMI.

Depression/well-being scores	C3		CRP		IL-6		Leptin		WBC		Neutrophils		Inflammatory score	
	β (R^2^)	*P*	β (R^2^)	*P*	β (R^2^)	*P*	β (R^2^)	*P*	β (R^2^)	*P*	β (R^2^)	*P*	β (R^2^)	*P*
**CES-D score**^a^														
Current smoker	0.018 (0.004)	**0.018**	0.791 (0.004)	**0.004**	0.756 (0.003)	**0.004**	0.723 (0.004)	**0.001**	1.195 (0.003)	0.106	1.204 (0.003)	**0.042**	0.184 (0.003)	**<0.001**
Heavy drinker	0.018 (0.001)	**0.018**	0.795 (0.000)	**0.004**	0.803 (0.000)	**0.002**	0.682 (0.000)	**0.002**	1.464 (0.000)	**0.037**	1.400 (0.000)	**0.014**	0.190 (0.000)	**<0.001**
Low-level physical activity	0.016 (0.015)	**0.04**	0.666 (0.015)	**0.018**	0.638 (0.015)	**0.017**	0.564 (0.014)	**0.01**	0.988 (0.015)	0.171	1.007 (0.014)	0.086	0.157 (0.014)	**<0.001**
Poor diet quality	0.017 (0.001)	**0.032**	0.661 (0.002)	**0.021**	0.771 (0.001)	**0.005**	0.688 (0.001)	**0.002**	1.413 (0.001)	0.053	1.376 (0.001)	**0.02**	0.181 (0.002)	**<0.001**
High BMI	0.016 (0.001)	**0.044**	0.742 (0.001)	**0.008**	0.753 (0.001)	**0.005**	0.621 (0.001)	**0.007**	1.369 (0.002)	0.053	1.314 (0.002)	**0.022**	0.185 (0.001)	**<0.001**
All lifestyle factors and BMI	0.014 (0.020)	0.111	0.469 (0.019)	0.12	0.513 (0.019)	0.071	0.566 (0.018)	**0.018**	0.543 (0.017)	0.494	0.701 (0.017)	0.27	0.137 (0.017)	**0.004**

**WHO-5 score**^a^														
Current smoker	−0.006 (0.001)	0.285	−0.466 (0.001)	**0.012**	−0.493 (0.001)	**0.006**	−0.339 (0.002)	**0.019**	−1.132 (0.001)	**0.023**	−0.928 (0.001)	**0.02**	−0.103 (0.001)	**<0.001**
Heavy drinker	−0.006 (0.004)	0.243	−0.488 (0.005)	**0.008**	−0.530 (0.005)	**0.003**	−0.330 (0.005)	**0.022**	−1.074 (0.005)	**0.023**	−0.917 (0.005)	**0.016**	−0.107 (0.005)	**<0.001**
Low-level physical activity	−0.005 (0.021)	0.358	−0.376 (0.020)	**0.046**	−0.414 (0.020)	**0.021**	−0.259 (0.020)	0.077	−0.754 (0.020)	0.118	−0.650 (0.020)	0.098	−0.079 (0.019)	**0.005**
Poor diet quality	−0.006 (0.001)	0.305	−0.344 (0.001)	0.072	−0.441 (0.001)	**0.016**	−0.308 (0.001)	**0.037**	−0.826 (0.001)	0.089	−0.726 (0.001)	0**.**064	−0.084 (0.001)	**0.003**
High BMI	−0.005 (0.001)	0.405	−0.443 (0.001)	**0.019**	−0.478 (0.001)	**0.007**	−0.284 (0.001)	0**.**064	−1.043 (0.001)	**0.028**	−0.863 (0.001)	**0.025**	−0.101 (0.001)	**0.001**
All lifestyle factors and BMI	−0.003 (0.027)	0.606	−0.221 (0.028)	0.267	−0.327 (0.027)	0.081	−0.216 (0.028)	0.171	−0.513 (0.028)	0.327	−0.491 (0.029)	0.24	−0.056 (0.027)	0.072

Abbreviations: C3: complement component 3; CES-D: Centre for Epidemiologic Studies Depression; CRP: c-reactive protein; IL-6: interleukin 6; WBC: white blood cell count.

Unstandardised β coefficients are shown. CRP, IL-6, leptin, WBC and neutrophils are log-transformed. Significant *p* shown in **bold**. R^2^ in models determined from dominance analyses. R^2^ in each model represents the variance explained by lifestyle factors and BMI, individually and all together.

a All models additionally adjusted for age, sex, education, anti-inflammatory medication use, type 2 diabetes, cardiovascular disease and cancer.

## 4 Discussion

In this cross-sectional study of 1824 middle-to older-aged men and women we hypothesised that lifestyle factors and BMI modulate the relationship between chronic-low grade inflammation and mental health. To test this, we first examined inflammatory biomarker relationships with CES-D depression and WHO-5 well-being scores. We observed associations with C3 (CES-D only) CRP, IL-6, leptin, WBCs, neutrophils and an inflammatory biomarker score (both CES-D and WHO-5). These relationships persisted following adjustment for demographic variables and health conditions. In analyses which included lifestyle factors, models which adjusted for low-level physical activity demonstrated greater attenuation than models which adjusted for other variables, with models including either smoking or alcohol use showing the least attenuation. However, models which adjusted for all lifestyle factors and BMI in general showed the greatest attenuation. Collectively, these findings suggest that the relationship between systemic low-grade inflammation and depressive symptoms and well-being may be largely explained by unhealthy lifestyle behaviours and increased adiposity.

Our observation that certain inflammatory biomarkers are related to psychological health is supported by the literature. In a study which examined subjects with a major depressive disorder, Luo et al. (2022) found that plasma concentrations of C3 were significantly higher than in healthy controls. In a systematic review of 56 studies, Orsolini et al. (2022) observed that, in most studies, higher blood CRP levels were associated with greater depression symptom severity and a worse response to treatment. Three meta-analyses studies have verified that people with a major depressive disorder show elevated serum/plasma IL-6 levels compared to people without depression (Dowlati et al., 2010; Howren et al., 2009; Liu et al., 2012). In two cross-sectional studies, Cernea et al. (2019) found that depressive and moderate-severe anxiety symptoms were associated with high leptin concentrations in type 2 diabetes patients. Depression and anxiety symptom relationships with WBCs have also been reported previously, with Schafiee et al. observing WBC levels to increase with increasing severity of symptoms of depression and anxiety among a sample of 3719 men (Shafiee et al., 2017).

Our suggestion that the relationship between systemic low-grade inflammation and depressive symptoms and well-being may be largely explained by lifestyle factors and BMI is also plausible based on previous studies. In research by our group, which examined core protective behaviours (never smoking, moderate alcohol intake, moderate to vigorous physical activity, a high-quality diet and a normal BMI), clear and independent inverse associations were observed between a five-component protective behaviour score and markers of chronic inflammation, as well as an adverse pro-atherogenic blood lipid profile (Millar et al., 2020, 2022a). Research has also demonstrated a relationship between lifestyle factors and depressed mood. In a cross-sectional analysis of 84,860 participants, Sarris et al. (2020) showed that physical activity and healthy diet were associated with less frequency of depressed mood while tobacco smoking was associated with a higher frequency. Previous work also indicates a cumulative protective effect of healthy lifestyle behaviours on mental health, as subjects with a greater number of healthy behaviours are less likely to report depressive symptoms (Adjibade et al., 2018; Maher et al., 2016). In research similar to the present study, which examined relationships between an inflammatory biomarker score and three scales assessing psychological resilience, depressive symptoms and mental well-being, Gialluisi et al. also found that associations with depressive symptoms and well-being persisted following adjustment for demographic variables and health conditions, but not for lifestyle factors (Gialluisi et al., 2020).

Among the lifestyle factors examined in the current study, it was found that models which adjusted for low-level physical activity demonstrated greater attenuation than models which adjusted for other lifestyle variables. Low-level physical activity was also found to explain a greater variance of both CES-D and WHO-5 scores. The therapeutic potential of exercise for patients with depression is well documented (Donoso et al., 2023), with studies consistently reporting a beneficial effect on depressive symptoms, stress and anxiety (Gialluisi et al., 2020; Hearing et al., 2016; Kandola et al., 2018; Vancampfort et al., 2018). The inhibitory effects of physical exercise on inflammation and microglial activation have been extensively reviewed and, consequently, a regulatory role for (neuro) inflammation in physical exercise-induced changes in depression has gained recent attention (Donoso et al., 2023). A reduction of the expression of toll-like receptors and pro-inflammatory cytokines in the hippocampus and brain cortex of rats fed with a high-fat diet and subjected to treadmill resistance exercise for five consecutive days has been reported (Kang et al., 2016). In ovariectomised mice, forced treadmill exercise for one week was found to reduce depression-like behaviours (as observed in a sucrose preference test and a forced swim test) by reducing IL-1β and IL-18 levels in the hippocampus (Wang et al., 2016). This suggests that endurance exercise ameliorates depression-like behaviour through inflammatory mechanisms (Donoso et al., 2023). In addition, fitness and exercise reduce leptin concentrations (Bobbert et al., 2012), elevated levels of which are implicated in the development of depression (Berk et al., 2013; Cernea et al., 2019; Pasco et al., 2008), as previously discussed.

It should be noted that there is still uncertainty regarding the relationship between chronic-low-grade inflammation and mental health. We modelled associations between biomarkers of systemic inflammation and depressive symptoms and well-being by hypothesising that the former influence the latter, although there is contrasting evidence on the directionality of this relationship (Gialluisi et al., 2020). As we found that unhealthy lifestyle factors and high BMI attenuate associations between biomarkers of inflammation and psychological health it must be considered that systemic inflammation may only be associated with depression and well-being because subjects with poorer mental health are less likely to engage in healthy lifestyle behaviours as a result of their symptoms. However, significant differences across psychological health score quartiles in this sample were only observed for low-level physical activity (both CES-D and WHO-5) and alcohol use (WHO-5 only). It has also been suggested that increased inflammation occurs only in a subset of subjects with depression and that they represent, at best, a depressive subtype (Miller, 2020). Further research is needed to parse apart the temporal relationship between chronic low-grade inflammation and mental health, and their shared risk factors (Mac Giollabhui, 2021).

This study has several strengths. The current work extends previous research by comparing CES-D depression and WHO-5 well-being score relationships with six biomarkers of chronic low-grade inflammation, and an inflammatory biomarker score, in a middle-to older-aged population. Other strengths include equal representation by sex (49% male) and the use of validated questionnaires to collect data.

However, a number of limitations should be considered. As previously discussed, the cross-sectional study design, which precludes drawing conclusions regarding the temporal direction of relationships, limits inference with respect to causality. In addition, although our assessment of mental health did not rely on participants' previous attendance to relevant health services, the use of self-reported questionnaires is subject to potential inaccuracies, recall and reporting bias. These include response sets such as social approval, social desirability and residual confounding arising from imprecise measurement of variables. Another limitation is that we did not examine other mental health disorders. As research suggests that inflammation is involved in multiple psychiatric conditions (Miller, 2020), further studies which assess these in the context of low-grade systemic inflammation and lifestyle factors are warranted. Additionally, ‘inflammageing’, a condition where older adults display increased levels of inflammatory biomarkers and progressively increasing cardiovascular risk, may distort the relationship between mental health and inflammatory biomarkers (Ferrucci and Fabbri, 2018). However, we attempt to mitigate these effects by controlling for age and cardiovascular disease history in regression analyses.

Finally, the generalisability of our findings may be limited. Ireland represents a generally ethnically homogeneous population (Cronin et al., 2008). In addition, previous research suggests that approximately 98% of Irish adults are registered with a GP and that, even in the absence of a universal patient registration system, it is possible to perform population-based epidemiological studies that are representative using our methods (Hinchion et al., 2002). However, our data were collected from a single primary care-based sample of middle-to older-aged adults which may not be representative of other populations and, therefore, further examination in other populations is suggested.

In conclusion, in agreement with previous research, our study demonstrates relationships between biomarkers of chronic-low grade inflammation and mental health. These associations survived adjustment for demographic variables and health conditions. However, models which additionally adjusted for lifestyle factors and BMI were attenuated. Consequently, these findings suggest that the relationship between systemic low-grade inflammation and depressive symptoms and well-being may be largely explained by unhealthy lifestyle behaviours and increased adiposity. Research on the complex inter-relationships between inflammation, lifestyle factors and mental health is important for public health, with a view to informing development of more effective population-based and individual (high-risk) prevention strategies, as studies on these can provide better insights into disease causation. Our findings suggest that modifications in certain lifestyle behaviours may help attenuate the relationship between low-grade inflammation and mental health and that lifestyle therapies could help reduce metabolic dysfunction and chronic disease risk; this may be of particular importance to older adults. However, as this was a cross-sectional study, further research is needed to further clarify the temporal relationship between chronic low-grade inflammation and mental health, and their shared risk factors.

## Funding

This research was funded by the Irish Health Research Board, grant number: HRC/2007/13. The funder had no role in the study design, data collection and analysis, decision to publish or preparation of the manuscript.

## CRediT authorship contribution statement

**Seán R. Millar:** Conceptualization, Data curation, Formal analysis, Methodology, Writing – original draft, Writing – review & editing. **Janas M. Harrington:** Data curation, Methodology, Project administration, Writing – review & editing, Funding acquisition. **Ivan J. Perry:** Project administration, Writing – review & editing, Funding acquisition. **Catherine M. Phillips:** Conceptualization, Funding acquisition, Project administration, Writing – review & editing.

## Declaration of competing interest

None.

## Data Availability

Data will be made available on request.
